# Compliance with American College of Chest Physicians (ACCP) recommendations for thromboembolic prophylaxis in the intensive care unit: a level I trauma center experience

**DOI:** 10.1186/s13037-021-00288-4

**Published:** 2021-03-25

**Authors:** Michael J. Waxman, Daniel Griffin, Erica Sercy, David Bar-Or

**Affiliations:** 1 Medical-Surgical Intensive Care Unit and Progressive Care Unit, Research Medical Center, Kansas City, MO USA; 2 Pulmonary and Critical Care, University of Missouri School of Medicine, Kansas City, MO USA; 3 Trauma Research Department, Research Medical Center, Kansas City, MO USA; 4 Injury Outcomes Network and Trauma Research LLC, 501 E. Hampden Avenue, Room 4-454, Englewood, Colorado 80113 USA

**Keywords:** Venous thromboembolism, Chemoprophylaxis, Intensive care unit

## Abstract

**Background:**

Recommendations are for nearly universal venous thromboembolism (VTE) prophylaxis in critically ill hospitalized patients because of their well-recognized risks. In those intensive care units (ICUs) where patient care is more uniformly directed, it may be expected that VTE prophylaxis would more closely follow this standard over units that are less uniform, such as open-model ICUs.

**Methods:**

This was a retrospective cohort study on all patients aged 18+ admitted to an open ICU between 6/1/2017 and 5/31/2018. Patients were excluded if they had instructions to receive comfort measures only or required therapeutic anticoagulant administration. Prophylaxis administration practices, including administration of mechanical and/or pharmacologic prophylaxis and delayed (≥48 h post-ICU admission) initiation of pharmacologic prophylaxis, were compared between patients admitted to the ICU by the trauma service versus other departments. Root causes for opting out of pharmacological prophylaxis were documented and compared between the two study groups.

**Results:**

One-hundred two study participants were admitted by the trauma service, and 98 were from a non-trauma service. Mechanical (98% trauma vs. 99% non-trauma, *P* = 0.99) and pharmacologic (54% vs. 44%, *P* = 0.16) prophylaxis rates were similar between the two admission groups. The median time from ICU admission to pharmacologic prophylaxis initiation was 53 h for the trauma service and 10 h for the non–trauma services (*P* ≤ 0.01). In regression analyses, trauma-service admission (odds ratio (OR) = 2.88, 95% confidence interval (CI) 1.21–6.83) and increasing ICU length of stay (OR = 1.13, 95% CI 1.05–1.21) were independently associated with pharmacologic prophylaxis use. Trauma-service admission (OR = 8.30, 95% CI 2.18–31.56) and increasing hospital length of stay (OR = 1.15, 95% CI 1.03–1.28) were independently associated with delayed prophylaxis initiation.

**Conclusions:**

Overall, the receipt of VTE prophylaxis of any type was close to 100%, due to the nearly universal use of mechanical compression devices among ICU patients in this study. However, when examining pharmacologic prophylaxis specifically, the rate was considerably lower than is currently recommended: 54% among the trauma services and 44% among non-trauma services.

## Background

Patients in the intensive care unit (ICU) are at increased risk for venous thromboembolism (VTE), the most common preventable cause of hospital death in the United States [1, 2], because of their risk factors, including immobility, frequency of endothelial injury from trauma and surgical procedures, and an increased likelihood of underlying disorders related to thrombophilic states [3–8]. Indeed, a previous study showed that almost all patients in a critical care unit had at least one major VTE risk factor, and a large proportion had multiple factors [9]. The frequencies of the two types of VTE, deep vein thrombosis and pulmonary embolism, in patients admitted to an ICU have been estimated at 5–31% and 0.4–2.3%, respectively [10–12].

Current recommendations of the American College of Chest Physicians (ACCP) include administration of pharmacologic VTE prophylaxis initiated as soon as possible upon ICU admission, unless the patient is at high risk of bleeding [13, 14]. Mechanical prophylaxis may alternatively be used if pharmacologic prophylaxis is deemed unsafe or specific contraindications exist [15–17]. Because VTE prophylaxis agents, including heparin, low molecular weight heparin (LMWH), fondaparinux, and direct oral anticoagulants [18, 19], have been shown to increase bleeding risk, choice and timing of administration should weigh the benefit of VTE prevention against a patient’s risk of bleeding [15–17, 20–23]. It is also vital that dosing of VTE prophylaxis is consistent, as missed doses significantly increase the risk of VTE [14, 24].

The structure of the ICU might be an important factor in VTE prophylaxis administration practices. Generally, ICUs are organized as either open, allowing any physician to admit, care for, and discharge patients, or closed, with those same functions relegated to only a few individuals, usually critical care physicians that are all part of the same group. Some ICUs have segments of their care closed to certain physicians, and other parts of the care are open. An example of this latter structure would be a single-group trauma service operating side-by-side with medical ICU patients whose attending physicians belong to a number of different groups.

Seven years before the current study, a quality improvement (QI) project at our tertiary care medical center’s medical and surgical ICUs assessed the prevalence of VTE prophylaxis treatment as various interventions were introduced. At the outset of the QI study, VTE prophylaxis, either pharmacologic or mechanical, was prescribed 42% of the time. With a widespread education campaign and placement of prophylaxis options on all ICU order sets, the rate increased to ~ 48%. Once a default order mandating the use of mechanical compression devices except where contraindicated (severe bilateral lower extremity trauma) was passed through the medical staff, the prophylaxis rate increased to over 90%. That default order was still present at the time of our current study.

The purpose of this study was to examine the VTE prophylaxis rates of patients in the ICU of this single medical center, compare pharmacologic prophylaxis to mechanical device rates, and compare those rates between the more uniformly managed single-group trauma-surgery service to patients cared for by non–trauma-surgeon physicians from multiple groups who practice in the ICU. We hypothesized that the “closed” trauma service would demonstrate a higher rate of pharmacologic VTE prophylaxis than a group of patients managed by multiple physicians from different groups practicing in the open-model medical-surgical ICU. Mechanical prophylaxis rates were also examined, but it was postulated that these rates would be high in both groups.

## Methods

This was a retrospective cohort study of patients age 18 years and older admitted to a 32-bed medical-surgical ICU (16-bed medical, 16-bed surgical) over the period 6/1/2017 to 5/31/2018. The study aim was to examine differences two primary outcomes—administration of pharmacologic prophylaxis (yes/no) and delayed initiation of pharmacologic prophylaxis administration (yes/no), defined as initiation ≥48 h after ICU admission—according to ICU admission service, categorized as trauma service vs. non–trauma service. Admission service was considered the primary exposure variable. Receipt of mechanical prophylaxis was also examined between the two study groups. All patients admitted to the ICU during the study period were eligible for study inclusion. Patients were excluded from the study if case notes indicated they were admitted to the ICU with instructions to receive comfort measures only or if they were admitted for a reason requiring therapeutic administration of anticoagulants (e.g., pre-existing DVT, PE, or acute myocardial infarction). The study population was comprised of a random sample of 100 patients admitted to the ICU by the trauma service and a random sample of 100 patients admitted by non–trauma-service physicians. Although the non–trauma-service physicians were from a number of different groups, their patients are referred to as a single group for analysis purposes. This study was approved by the Institutional Review Board at the participating facility and received a waiver of HIPAA authorization and informed consent. Study data were obtained from the facility’s trauma registry and patient electronic medical records.

The trauma service has a daily program during trauma-physician–led multidisciplinary rounds of reviewing mechanical and pharmacologic prophylaxis on all ICU trauma patients. A monthly trauma QI program reviews any patients who did not receive pharmacologic prophylaxis, had missing doses of prophylactic anticoagulants, or lacked mechanical prophylaxis and subsequently reports out to the monthly Trauma Morbidity and Mortality meeting attended by all trauma surgeons and various other consulting physicians and administrative personnel.

During the study period, an automated order was in place for mechanical prophylaxis to be initiated on all patients upon admission to the ICU. Ordering providers had to opt-in for pharmacologic prophylaxis at their discretion. The mechanical prophylaxis method used in the ICU was sequential compression devices, and the pharmacologic prophylaxis types administered during the study were enoxaparin, heparin, and apixaban.

Clinical descriptors collected included age, gender, race, service admitting to the ICU (trauma service or non-trauma service), primary hospital admission diagnosis, including further categorization into traumatic injury versus any other diagnosis, comorbidities (hypertension, smoking, diabetes, liver disease/disorder, obesity, history of VTE, kidney disease/disorder, cancer), admission Glasgow Coma Scale (GCS) score, pre-admission anticoagulants, and any clotting- or bleeding-related complications occurring during the ICU stay (e.g., DVT, PE, intracranial hemorrhage, surgical site bleed). Detailed information about prophylaxis administration was also collected: administration of VTE prophylaxis (mechanical or pharmacologic) during the ICU stay; start and stop times of prophylaxis; type of prophylaxis administered (mechanical, pharmacologic, or both); type of pharmacologic prophylaxis administered, if applicable (enoxaparin, heparin, or apixaban); sequence of prophylaxis administration (mechanical to pharmacologic, as well as various types of pharmacologic to each other); and whether a patient experienced any interruptions in prophylaxis administration during which they received no prophylaxis of any type after prior initiation. Additionally, if documented in the electronic medical record, the reasons for any delayed initiation or interruption of prophylaxis were recorded.

Patient demographics and clinical characteristics were described in the ICU population, and differences between trauma-service and non–trauma-service ICU admissions were evaluated using chi-square tests for categorical variables and Wilcoxon rank-sum tests for continuous variables because of non-normal distribution. Prophylaxis administration practices were described in the ICU, and these practices were compared between trauma-service and non–trauma-service admissions using chi-square tests. Rates of clotting- and bleeding-related complications were also described in the ICU population and compared between trauma-service and non–trauma-service admissions using chi-square tests. Unadjusted and adjusted logistic regression models were used to identify factors associated with two outcomes: administration of pharmacologic prophylaxis and delayed pharmacologic prophylaxis initiation (≥48 h after ICU admission). Adjusted models evaluated ICU admission service (trauma service vs. non–trauma service) as the primary exposure variable and additionally considered the following variables for potential inclusion as covariates: patient age, sex, race, hospital length of stay, ICU length of stay, pre-admission anticoagulants, comorbidities, and primary hospital admission diagnosis. Stepwise selection with entry criteria of α = 0.20 and exit criteria of α = 0.05 was used to determine the final adjusted model.

## Results

A total of 200 patients were included in this study, of which the trauma service oversaw 102, and 98 of which were overseen by non–trauma-service physicians (Table 1). Of the non–trauma-service admissions, 42 (43%) were admitted from the medical department, 22 (22%) were from neurosurgery, 21 (21%) were from cardiology, 7 (7%) were non-neurosurgical surgery patients, 3 (3%) were from nephrology, 1 (1%) was from burn services, 1 (1%) was from pulmonology, and 1 (1%) was from another non-surgical department.

**Table 1:** Characteristics of the intensive care unit patient population

	All	Trauma service	Non–trauma service	P
	*n* = 200	*n* = 102	*n* = 98	
*Patient demographics*				
Age, years, median (IQR)	59 (39–74)	52 (29–65)	65 (54–75)	**< 0.01**
Sex				0.08
Male	138 (70%)	75 (76%)	63 (64%)	
Female	59 (30%)	24 (24%)	35 (36%)	
Race				0.21
White	126 (63%)	62 (61%)	64 (65%)	
Black	64 (32%)	37 (36%)	27 (28%)	
Other/unknown	10 (5%)	3 (3%)	7 (7%)	
*Clinical descriptors*				
Hospital length of stay, days, median (IQR)	6 (3–10)	6 (2–10)	5 (3–9)	0.47
ICU length of stay, days, median (IQR)	3 (1–5)	3 (2–4)	3 (1–6)	0.94
Pre-admission anticoagulants	34 (26%)	13 (20%)	21 (32%)	0.14
Comorbidities				
Hypertension	82 (42%)	34 (34%)	48 (49%)	0.04
Current smoker	33 (17%)	27 (27%)	6 (6%)	**< 0.01**
Diabetes	25 (13%)	11 (11%)	14 (14%)	0.50
Liver disease	12 (6%)	9 (9%)	3 (3)	0.08
Obesity	12 (6%)	1 (1%)	11 (11%)	**< 0.01**
History of VTE	4 (2%)	1 (1%)	3 (3%)	0.31
Chronic kidney disease	3 (1.5%)	0 (0%)	3 (3%)	0.12
Cancer	2 (1%)	0 (0%)	2 (2%)	0.25
Presence of traumatic injury				**< 0.01**
Yes	110 (55%)	95 (93%)	15 (15%)	
No	90 (45%)	7 (7%)	83 (85%)	
Primary hospital admission diagnosis				**< 0.01**
Head or facial injury	57 (29%)	43 (43%)	14 (15%)	
Injuries of the thorax, abdomen, or neck	39 (20%)	38 (38%)	1 (1%)	
Cardiovascular disease	21 (11%)	2 (2%)	19 (20%)	
Extremity injuries, including hip injuries	15 (8%)	14 (14%)	1 (1%)	
Headache, fatigue, altered mental state	13 (7%)	0 (0%)	13 (14%)	
Musculoskeletal, connective tissue, nervous system diseases, convulsions	9 (5%)	0 (0%)	9 (9%)	
Respiratory condition or trouble breathing	8 (4%)	1 (1%)	7 (7%)	
Neoplasm	7 (4%)	0 (0%)	7 (7%)	
Chest pain	7 (4%)	0 (0%)	7 (7%)	
Digestive issues or abdominal pain	5 (3%)	0 (0%)	5 (5%)	
Asphyxiation, burns, poisoning	4 (2%)	1 (1%)	3 (3%)	
Complications of prosthetic devices, implants or grafts	2 (1%)	0 (0%)	2 (2%)	
Endocrine, nutritional, or metabolic diseases	2 (1%)	0 (0%)	2 (2%)	
Infectious disease	2 (1%)	0 (0%)	2 (2%)	
Rash	2 (1%)	0 (0%)	2 (2%)	
Unspecified hemorrhage, unspecified pain	2 (1%)	1 (1%)	1 (1%)	

Bold indicates statistically significant results at a threshold of *P* ≤ 0.05. IQR, interquartile range; *ICU* intensive care unit, *VTE* venous thromboembolism

The median (IQR) age of the patient population was 59 (39–74) years, and 138 (70%) were male (Table 1). The median (IQR) total hospital length of stay was 6 (3–10) days, and the median (IQR) ICU length of stay was 3 (1–5) days. Twenty-six percent (*n* = 34) of patients were on anticoagulants before hospital admission. Forty-two percent (*n* = 82) of patients had pre-existing hypertension, 17% (*n* = 33) were current smokers, and 13% (*n* = 25) had diabetes. The most common reasons for hospital admission were traumatic injury (55%), cardiovascular disease (11%), and headache, fatigue, or altered mental state (7%). The majority (93%, *n* = 95) of patients admitted to the ICU by the trauma service had traumatic injuries, and 15 (15%) patients admitted by a non–trauma-service physician had traumatic injuries.

The overall VTE prophylaxis rate in the ICU, including mechanical and pharmacologic, was 99% (101 of 102) for the trauma service and 100% (98 of 98) for the non–trauma-service patients (*P* = 0.99, Table 2). The overall administration rate of pharmacologic prophylaxis was 49% (*n* = 98 of 200), and the mechanical prophylaxis administration rate was 99% (199 of 200). The rates of mechanical prophylaxis were 98% (100 of 102) in the trauma service and 99% (97 of 98) in the non–trauma-service group (*P* = 0.99). Pharmacologic prophylaxis was prescribed more often in the trauma service group, at 54% (55 of 102), but this was not statistically significantly different (*P* = 0.16) than the 44% (43 of 98) in non–trauma-service patients. When evaluating each prophylaxis type, mechanical prophylaxis alone (without initiation of pharmacologic prophylaxis) was utilized more often in the non–trauma-service group, at 56%, but this was not statistically significantly higher than in the trauma-service group (45%, *P* = 0.12). Administration of pharmacologic prophylaxis alone without any administration of mechanical prophylaxis occurred in 1% for both groups. The trauma service had more patients that received a combination of both pharmacologic and mechanical prophylaxis during their ICU stay, at 53%, but this was not significantly higher than the non–trauma-service group (43%, *P* = 0.15).

**Table 2:** Venous thromboembolism prophylaxis administration practices in the intensive care unit

	All	Trauma service	Non–trauma service	*P*
	*n* = 200	*n* = 102	*n* = 98	
VTE prophylaxis administered	199 (99%)	101 (99%)	98 (100%)	0.99
Pharmacologic prophylaxis administered	98 (49%)	55 (54%)	43 (44%)	0.16
Mechanical prophylaxis administered	197 (98%)	100 (98%)	97 (99%)	0.99
VTE prophylaxis type				
Mechanical only	101 (51%)	46 (45%)	55 (56%)	0.12
Pharmacologic only	2 (1%)	1 (1%)	1 (1%)	0.99
Both	96 (48%)	54 (53%)	42 (43%)	0.15
Pharmacologic prophylaxis administered				**< 0.01**
Enoxaparin only	71 (73%)	52 (95%)	19 (45%)	
Heparin only	17 (18%)	3 (5%)	14 (33%)	
Apixaban only	1 (1%)	0 (0%)	1 (2%)	
Heparin, followed by enoxaparin	4 (4%)	0 (0%)	4 (10%)	
Enoxaparin, followed by heparin	2 (2%)	0 (0%)	2 (5%)	
Enoxaparin, followed by apixaban	1 (1%)	0 (0%)	1 (2%)	
Simultaneous heparin and enoxaparin	1 (1%)	0 (0%)	1 (2%)	
Interruption of pharmacologic prophylaxis after initiation	10 (10%)	5 (9%)	5 (12%)	0.74
Interruption of enoxaparin	8 (10%)	5 (10%)	3 (11%)	0.99
Interruption of heparin	2 (8%)	0 (0%)	2 (9%)	0.99
Time between admission and pharmacologic prophylaxis initiation (hours), median (IQR)	23 (8–76)	53 (15–107)	10 (2–25)	**< 0.01**
Admission to enoxaparin initiation (hours), median (IQR)	30 (14–77)	53 (15–95)	17 (4–25)	**< 0.01**
Admission to heparin initiation (hours), median (IQR)	7 (0–29)	131 (17–188)	4 (0–21)	**0.03**
Admission to apixaban initiation (hours), median (IQR)	2 (2–2)	–	2 (2–2)	–
Reasons for non-administration or interruption of prophylaxis				
Holding for procedure^a^	26 (46%)	14 (67%)	12 (33%)	**0.03**
Active bleed	17 (30%)	8 (38%)	9 (25%)	0.30
Recent TBI (≤48 h)	17 (30%)	12 (57%)	5 (14%)	**< 0.01**
High bleeding risk	3 (5%)	0 (0%)	3 (8%)	0.30
Low platelet count (< 50,000)	3 (5%)	1 (5%)	2 (6%)	0.90
Epidural catheter	2 (4%)	2 (10%)	0 (0%)	0.13
Intracranial pressure monitor	1 (2%)	1 (5%)	0 (0%)	0.37
Other^b^	13 (29%)	4 (19%)	9 (25%)	0.75
No documented reason for treatment holding or interruption	9 (16%)	0 (0%)	9 (25%)	**0.01**

Bold indicates statistically significant results at a threshold of *P* ≤ 0.05. *VTE* venous thromboembolism, *IQR* interquartile range, *TBI* traumatic brain injury. ^a^Procedures resulting in prophylaxis holds: ankle fixation, brain biopsy, bronchoscopy, burr hole, coronary artery bypass graft, cardiac repair, craniotomy, endovascular coiling, image-guided percutaneous abscess drainage, inferior vena cava filter placement, massive transfusion protocol, open reduction internal fixation, odontoid screw fixation, removal of epidural catheter, sacroiliac screw fixation, tracheostomy, ulnar repair. ^b^Other documented reasons for holding: cardiac issues, change in diagnosis, undefined contraindication to pharmacologic prophylaxis, code, diagnosis changed to stroke, INR not reaching subtherapeutic goal, open gastrostomy tube, patient religious objections, transfer to another hospital, and patient-requested withdrawal of care

Of the patients who were administered pharmacologic prophylaxis, most received enoxaparin only (73%, *n* = 71), and 18% (*n* = 17) received heparin only. One patient (1%) received apixaban only, and the remainder of the patients (*n* = 8) received some combination of these medications. The most marked difference (*P* < 0.01) between trauma-service and non–trauma-service admissions was that almost all patients admitted to the ICU by the trauma service received enoxaparin only (95%, *n* = 52), with the remainder receiving heparin only (5%, *n* = 3), whereas among non–trauma-service admissions, just 45% (*n* = 19) received enoxaparin only, and 33% (*n* = 14) received heparin only. The remainder of the non–trauma-service admissions received apixaban only (2%, *n* = 1) or a combination of medications (e.g., enoxaparin followed by heparin, simultaneous enoxaparin and heparin; *n* = 9). Although not explicitly delineated, higher utilization of heparin in the non–trauma-service group may be related to common reasons heparin is used over LMWH, such as renal dysfunction.

After the initiation of pharmacologic prophylaxis, 10% of the total cohort had interruptions. Trauma-service patients experienced an interruption 9% of the time, and non–trauma-service patients experienced an interruption 12% of the time (*P* = 0.74). When evaluating the two major types of pharmacologic prophylaxis (enoxaparin and heparin), the interruption rates of both drugs were similar between both the trauma-service and non–trauma-service groups (*P* = 0.99).

When assessing delays in the initiation of pharmacologic prophylaxis, the median delay for the two groups combined was 23 h after ICU admission. The median initiation delay for the trauma service was 53 h, and the median delay for the non–trauma-service group was 10 h (*P* ≤ 0.01). Delays in initiation were additionally evaluated individually for enoxaparin and heparin. Enoxaparin was initiated a median of 30 h after ICU admission among the entire study population. Among patients overseen by the trauma service, enoxaparin was initiated a median of 53 h after ICU admission, and the median for the non–trauma-service group was 17 h (*P* ≤ 0.01). Heparin was initiated a median of 7 h after ICU admission among the entire study population. The median initiation time among the trauma service was 131 h after ICU admission, and the median among the non–trauma-service group was 4 h (*P* = 0.03).

The most common reason for holding prophylaxis, defined as either administering no prophylaxis or interrupting it after initiation, was discontinuation before a surgical procedure (46% of those with interrupted or no prophylaxis, *n* = 26), followed by recent head trauma with fracture or brain injury (referred to here as traumatic brain injury (TBI) within the previous 48 h) (30%, *n* = 17) and the presence of an active bleed (30%, *n* = 17). Nine patients (16%) had no documented reason for non-administration or interruption; all these patients were admitted to the ICU by non–trauma-service physicians. Complications related to VTE prophylaxis administration, including DVT, PE, and bleeding, were rare in the study population. One trauma-service admission (1%) experienced a PE while in the ICU, and one non–trauma-service admission (1%) developed severe liver dysfunction.

In adjusted logistic regression analyses using receipt of pharmacologic prophylaxis as the outcome, trauma-service admitting department (odds ratio (OR) = 2.88, 95% confidence interval (CI) 1.21–6.83) and increasing ICU length of stay (OR = 1.13, 95% CI 1.05–1.21) were significantly independently associated with the outcome (Table 3). When evaluating delays in pharmacologic initiation, defined here as initiation > 48 h after ICU admission, adjusted logistic regression analyses showed that trauma-service admission (OR = 8.30, 95% CI 2.18–31.56) and increasing hospital length of stay (OR = 1.15, 95% CI 1.03–1.28) were significantly associated with this outcome.

**Table 3:** Univariate and independent associations with pharmacologic prophylaxis administration in the intensive care unit

	Pharmacologic prophylaxis administered		Substantially delayed pharmacologic initiation^a^	
	Unadjusted OR (95% CI)	Adjusted OR (95% CI)	Unadjusted OR (95% CI)	Adjusted OR (95% CI)
ICU admitting service				
Non-trauma service	Ref	Ref	Ref	Ref
Trauma service	1.50 (0.86–2.61)	**2.88 (1.21–6.83)**	**7.96 (2.89–21.95)**	**8.30 (2.18–31.56)**
Age	0.99 (0.97–1.00)		0.98 (0.96–1.00)	
Sex				
Male	Ref		Ref	
Female	0.80 (0.43–1.47)		0.61 (0.24–1.58)	
Race				
White	Ref		Ref	
Black	1.46 (0.80–2.66)		1.29 (0.55–3.04)	
Other/unknown	1.75 (0.47–6.46)		0.34 (0.04–3.14)	
Hospital length of stay	**1.05 (1.01–1.08)**		**1.04 (1.00–1.09)**	**1.15 (1.03–1.28)**
ICU length of stay	**1.10 (1.02–1.19)**	**1.13 (1.05–1.21)**	1.06 (0.99–1.13)	
Pre-admission anticoagulants	0.59 (0.27–1.31)		3.00 (0.89–10.07)	
Comorbidities				
Hypertension	1.16 (0.66–2.05)		0.73 (0.31–1.70)	
Smoking	1.23 (0.58–2.60)		2.13 (0.70–6.46)	
Diabetes	0.92 (0.40–2.14)		0.80 (0.22–2.88)	
Liver disease	1.01 (0.31–3.25)		0.81 (0.14–4.68)	**0.07 (0.01–0.92)**
Obesity	1.45 (0.44–4.72)		0.10 (0.01–2.13)	
History of VTE	0.33 (0.03–3.23)		0.53 (0.01–50.75)	
Kidney disease	0.14 (0.01–4.34)		–	
Cancer	0.20 (0.01–8.24)		–	
Traumatic injury	1.19 (0.68–2.07)		**7.44 (2.70–20.47)**	
Head injury	0.24 (0.12–0.48)	0.22 (0.08–0.59)	**5.28 (1.52–18.36)**	

Bold indicates statistically significant results at a threshold of *P* ≤ 0.05. ^a^Substantially delayed initiation of pharmacologic VTE prophylaxis was defined as initiation > 48 h after admission to the ICU. *OR* odds ratio, *95% CI* 95% confidence interval, *ICU* intensive care unit, *VTE* venous thromboembolism

## Discussion

This study found that in a single, open medical-surgical ICU in a tertiary care non-academic medical center, pharmacologic prophylaxis was not administered at rates consistent with ACCP recommendations by either the uniformly managed trauma service or the non-trauma service. Rates were higher for pharmacologic prophylaxis ordered on patients under the care of the trauma service. Although there were no significant differences in pharmacologic prophylaxis rates between admitting groups, both hovering around 50%, adjusted regression analyses showed trauma-service admission to be a significant predictor of pharmacologic prophylaxis administration. Delayed initiation of pharmacologic prophylaxis was, however, more likely in the trauma group. These results imply that after accounting for patient-level differences between the groups that affected the likelihood of prophylaxis administration (e.g., more patients with TBI in the trauma-service group), patients admitted to the ICU by the trauma service were more likely to receive pharmacologic prophylaxis during their ICU stay.

Because of the lower-than-recommended administration rates across both groups, we further investigated reasons for non-administration or interruption of prophylaxis. The trauma service documented a specified reason 100% of the time if there was no pharmacologic therapy or an interruption of therapy. Although the duration of the interruption is unknown, the documented reasons for interruption were consistent with standard practice. This differed from the non-trauma patient group, in which 25% of the instances of non-administration or interruption had no documented reason.

Prior to the study period reported here, the participating facility implemented an automatic order for mechanical prophylaxis on every patient admitted to the ICU, aiming to increase the percentage of patients receiving some type of VTE prophylaxis. This was based on a QI project that demonstrated a < 50% rate for any VTE prophylaxis in the ICU. It was not until the automatic ordering of mechanical prophylaxis was instated that prophylaxis rates approached 100%. Not surprisingly, the mechanical prophylaxis rate in the current study was 99% among the trauma-service patients and 100% among the non–trauma-service patients. Use of mechanical prophylaxis is part of daily QI on nursing rounds, with “fall outs” reported to attending physicians for rectification.

Pharmacologic VTE prophylaxis is both indicated for ICU patients and more effective than mechanical prophylaxis. Translating this recommendation into regular practice is challenging. Improving the practice in the trauma service is part of the QI process that has been established. Non-administration and interruptions of pharmacologic prophylaxis are reviewed regularly on all trauma-service patients. Improving the practice of pharmacologic prophylaxis among non–trauma-service patients will likely require a different approach. One approach would be to close the non-trauma part of the ICU to the non-trauma pulmonary intensivist service. Another approach may be similar to the mechanical prophylaxis order set: making pharmacologic prophylaxis a default, held only for specific contraindications.

One limitation of this study was a small sample size. Because the outcomes of VTE and bleeding complications are infrequent, the sample size did not allow for conclusions about prophylaxis administration and associated outcomes. However, the sample size was sufficient to allow a description of prophylaxis administration practices overall in the ICU and evaluate whether these practices differed between patients overseen by the trauma service and those overseen by non–trauma-service physicians. An additional limitation was the grouping of ICU admissions into those patients admitted to the ICU by the trauma service compared to those admitted by non–trauma-service hospital departments. Patients admitted to the ICU by the trauma service were relatively similar in terms of the primary reason for hospital admission: 93% of trauma-service admissions were admitted for traumatic injuries. Patients admitted by non–trauma-service departments had wide-ranging reasons for hospital admission, including traumatic injury, neoplasm, infectious disease, and chest pain, among many others. Future work may consider using a more similar patient population as a comparison group.

## Conclusions

Overall, VTE prophylaxis of any type was high in both the trauma and non-trauma service. However, both services underused pharmacologic prophylaxis specifically. Delays in initiation of pharmacologic prophylaxis were more common in patients admitted to the ICU by the trauma service. Despite these delays, rates of patients receiving pharmacologic prophylaxis were higher among those patients admitted and overseen by the trauma service. Additionally, the trauma service commonly documented their reasons for holding pharmacologic prophylaxis and the non-trauma service was less likely to follow this practice.

## Data Availability

The datasets used and/or analyzed during the current study are available from the corresponding author on reasonable request.

## Abbreviations

ICU: Intensive care unit
VTE: Venous thromboembolism
ACCP: American College of Chest Physicians
DVT: Deep venous thrombosis
PE: Pulmonary embolism
LMWH: Low molecular weight heparin
QI: Quality improvement
GCS: Glasgow Coma Scale
IQR: Interquartile range
TBI: Traumatic brain injury
OR: Odds ratio
CI: Confidence interval
